# Thermodynamics and kinetics of amphotericin B self-association in aqueous solution characterized in molecular detail

**DOI:** 10.1038/srep19109

**Published:** 2016-01-08

**Authors:** Joanna Zielińska, Miłosz Wieczór, Tomasz Bączek, Marcin Gruszecki, Jacek Czub

**Affiliations:** 1 Department of Pharmaceutical Chemistry, Medical University of Gdańsk, Gdańsk, Poland; 2 Department of Physical Chemistry, Gdańsk University of Technology, Gdańsk, Poland; 3 Department of Radiological Informatics and Statistics, Medical University of Gdańsk, Gdańsk, Poland

## Abstract

Amphotericin B (AmB) is a potent but toxic drug commonly used to treat systemic mycoses. Its efficiency as a therapeutic agent depends on its ability to discriminate between mammalian and fungal cell membranes. The association of AmB monomers in an aqueous environment plays an important role in drug selectivity, as oligomers formed prior to membrane insertion – presumably dimers – are believed to act differently on fungal (ergosterol-rich) and mammalian (cholesterol-rich) membranes. In this work, we investigate the initial steps of AmB self-association by studying the structural, thermodynamic and spectral properties of AmB dimers in aqueous medium using molecular dynamics simulations. Our results show that in water, the hydrophobic aggregation of AmB monomers yields almost equiprobable populations of parallel and antiparallel dimers that rapidly interconvert into each other, and the dipole-dipole interaction between zwitterionic head groups plays a minor role in determining the drug’s tendency for self-aggregation. A simulation of circular dichroism (CD) spectra indicates that in experimental measurements, the signature CD spectrum of AmB aggregates should be attributed to higher-order oligomers rather than dimers. Finally, we suggest that oligomerization can impair the selectivity of AmB molecules for fungal membranes by increasing their hydrophobic drive for non-specific membrane insertion.

Due to the lack of effective antifungal agents, deep-seated mycoses are a serious problem in medicine^1^. One of the most potent antifungal drugs used to treat systemic fungal infections is Amphotericin B (AmB, Fig. 1) – a polyene macrolide antibiotic, whose efficiency comes, however, at the cost of high toxicity^2^. To date, any attempts to introduce rationally designed safer AmB derivatives to the drug market have failed, largely because the mechanism of action of AmB at the molecular level remains unclear^3^,^4^,^5^,^6^.

Well-established as a membrane-active drug, AmB has been experimentally shown to form transmembrane channels^7^,^8^,^9^,^10^ that mediate the efflux of essential cellular components and, consequently, lead to cell death. It has also been proposed that the therapeutic effect of AmB stems from its higher efficiency in permeabilizing fungal cell membranes with ergosterol than mammalian membranes with cholesterol^11^,^12^,^13^,^14^,^15^,^16^,^17^. Yet, the detailed role of membrane sterols in modulating the cytotoxic activity of AmB is still unknown^18^,^19^.

Several previous studies linked the selectivity of AmB for the fungal membranes to its self-association properties^20^,^21^. Interestingly, self-aggregation in the water phase prior to membrane insertion was suggested to contribute to this selectivity. Indeed, the amphiphilic AmB molecules readily form aggregates even in micromolar (therapeutic) concentrations. Based on correlation between the spectral and membrane-permeabilizing properties^22^, as well as surface pressure measurements^23^, it was suggested that AmB monomers permeabilize only ergosterol-containing membranes, while the side effects are due to the interactions of self-associated forms, possibly dimers, with cholesterol-rich membranes. Similarly, Lambing *et al.* and Huang *et al.* suggested that dimerization of AmB molecules is a prerequisite for their insertion into cholesterol-rich membranes^16^,^24^.

Notably, the spontaneous self-aggregation of AmB in aqueous medium is also highly relevant for an alternative mode of action of the drug, put forward by Gray *et al.*^4^ According to this hypothesis, AmB molecules form large, sponge-like extramembranous aggregates that preferentially bind ergosterol but not cholesterol molecules. By extracting sterols from the fungal cell membrane, the aggregates could affect membrane physiology, eventually triggering cell death.

The self-aggregation of AmB was mostly studied by means of molecular spectroscopy. In aqueous media, the optical absorption as well as circular dichroism (CD) spectra depend qualitatively on the drug concentration. As the concentration exceeds 0.1 μM, a strong distinct signal typically ascribed to an exciton doublet arises in the CD spectrum, indicating the formation of oligomers^22^,^25^,^26^. Specifically, several studies have focused on the understanding of the AmB dimerization process as a possible early stage of aggregation in water. AmB dimers have been investigated by fluorescence spectroscopy^27^,^28^ and time-resolved single-molecule spectroscopy^29^. While these studies reported the coexistence of two dimer geometries, in which the antibiotic molecules are aligned either in a parallel or antiparallel manner, little is known about the equilibrium between them, their structural dynamics and formation kinetics. In particular, it remains unclear whether dimers alone can yield the signature CD spectrum.

In this study, we present a detailed analysis of the self-association of amphotericin B in an aqueous environment. Using molecular dynamics, we quantitatively assessed the thermodynamic stability of various dimer geometries and the associated kinetic parameters, and found that AmB dimers exist in several stable arrangements, with a roughly equiprobable population of parallel and antiparallel arrangements and a sub-microsecond rate of flipping between them. This is due to the association being almost entirely hydrophobic in nature, and mostly unaffected by the large dipole moment of the zwitterionic head group. By assigning each geometry its signature circular dichroism spectrum and confronting the spectra with existing experimental data, as well as by simulating the spontaneous association of dimers, we speculate on the structural properties of higher-order agglomerates that form even at micromolar concentrations. We also discuss a possible impact of the hydrophobic solvent-exposed surface area of oligomers on the specificity of drug-membrane interactions, and propose a plausible explanation for the toxic side effects of AmB.

## Results & Discussion

### AmB dimers form at submicromolar concentrations

To study the structure and stability of AmB dimers in an aqueous environment, we computed the free energy profile for the dimerization process, as a function of the separation distance between the polyene chromophores of two AmB molecules. As can be seen in Fig. 2A, the obtained profile is characterized by a broad minimum that corresponds to the formation of the dimer. From the profile, we calculated the standard free energy of dimerization ΔG°_d_ (as described in Methods) to be −7.1 kcal/mol. Based on this value, the onset of dimerization (10% of AmB molecules in dimeric form) was estimated to occur at the concentration of 0.41 μM, consistently with experimental measurements that reported the existence of monomers in concentrations up to 0.1 μM^14^. At 1 μM, 19% of AmB molecules were estimated to exist in the dimeric form, assuming no higher-order aggregation. This suggests that in therapeutically relevant concentrations, dimers might indeed constitute a significant fraction of AmB molecules, contributing to the toxicity towards cholesterol-rich membranes as proposed in the literature^16^,^22^,^23^,^24^.

Within the broad free energy basin, one can distinguish two local minima (ca. −5.5 kcal/mol with respect to the monomeric state) located at 4.5 and 6.5 Å and separated by a relatively low (1.5 kcal/mol) barrier, indicative of structural diversity of the AmB dimers. Notably, the location of minima matches well the interchromophore distance as determined experimentally by Starzyk *et al.* on the basis of exciton splitting theory^29^, where the closer minimum was attributed to a parallel and the farther to an antiparallel arrangement of AmB molecules.

**Figure 1:**
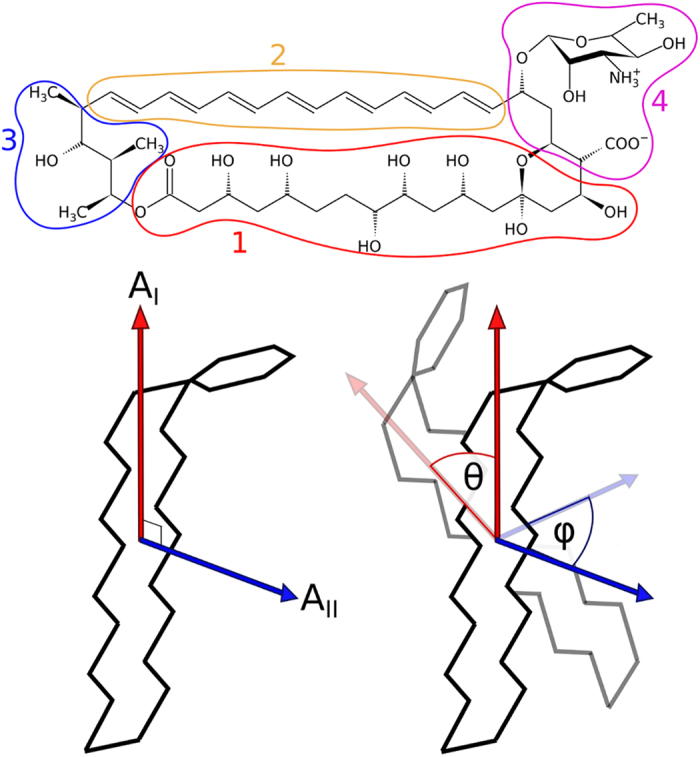
Molecular structure of AmB and angular reaction coordinates used in the study. Upper panel: Chemical structure of Amphotericin B divided into four parts: polyol chain (1), polyene chain (2), hydrophilic tail (3) and the polar head encompassing the mycosamine residue and the carboxyl moiety (4). Bottom panel: Schematic representation of the two principal axes and angles between them, used as orientation parameters to describe dimer geometry.

**Figure 2:**
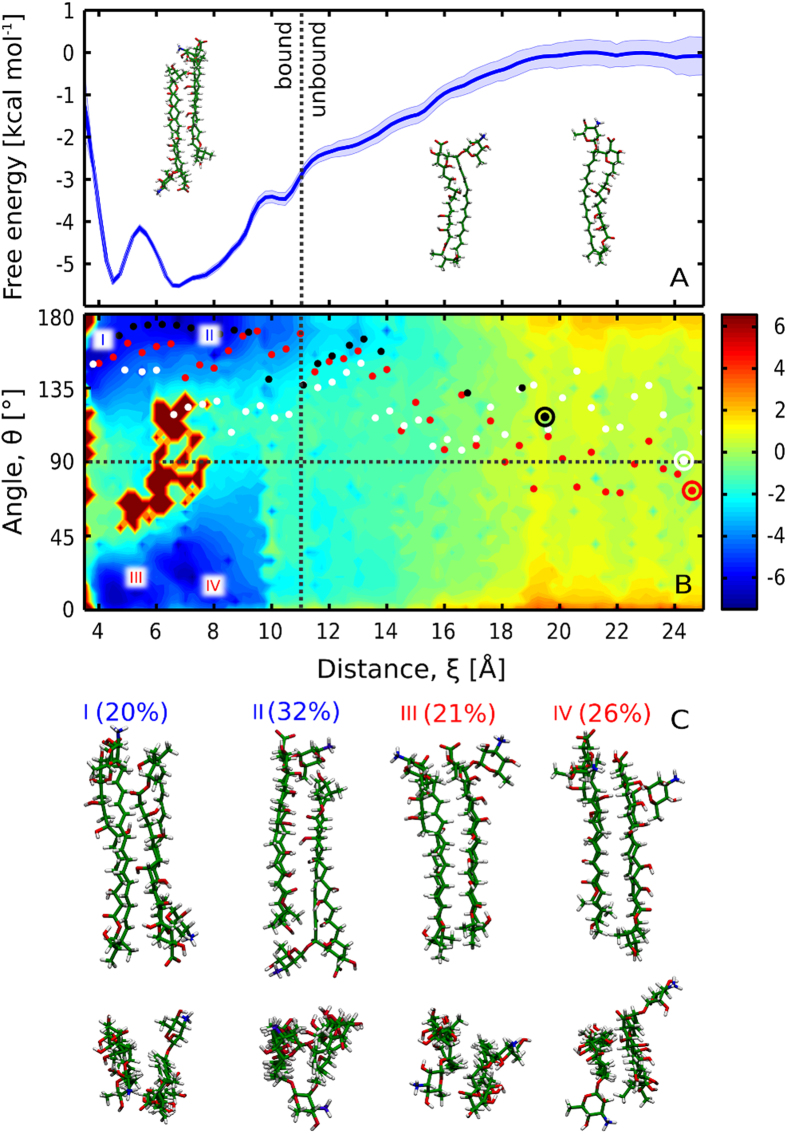
Dimerization free energy profiles of AmB molecules. (**A**) The 1D profile reveals that AmB association is barrierless, however a barrier separates two subpopulations of dimers. (**B**) The 2D profile indicates the existence of four free energy basins, marked as I-IV. Red, white and black dots correspond to the spontaneous association trajectories projected onto the reaction coordinate plane. The shape of the 2D free energy surface prompted us to set the boundary between the bound (dimeric) and unbound state of two monomers at ξ = 11 Å, as indicated with a dotted vertical line. **(C):** Representative structures corresponding to individual free energy basins I-IV; see also Fig. S1.

To test this assignment against our MD results, for each simulation frame we calculated the angle θ between the longest principal axes of two AmB molecules (marked as AI in Fig. 1, bottom panel). By reweighing the umbrella sampling data to recover the unbiased Boltzmann distribution, we obtained the free energy profile as a function of both the interchromophore distance ξ and the angle θ, as shown in Fig. 2B. It can be seen that the distinction of parallel and antiparallel geometries does not directly allow to resolve the nature of the two minima mentioned above, as in the 2D profile two new minima appear for both parallel and antiparallel arrangements (marked as I-IV in Fig. 2B, and illustrated in Fig. 2C). Indeed, we found that the average values of ξ for parallel (θ < 90°) and antiparallel (θ > 90°) dimers are similar (5.6 and 6.0 Å, respectively).

### Parallel and antiparallel dimer geometries are almost equiprobable

As it remains unclear which dimer arrangement prevails under physiological conditions, we also used the 2D profile in Fig. 2B to compute the relative probability of the two geometries, and found that the formation of parallel and antiparallel dimers is almost equally probable, with only a slight (6%) difference in favor of the antiparallel geometry. The four minima, I-IV, correspond to 20, 32, 21 and 26% of the overall population of dimers (in respective order). This indicates an almost equiprobable distribution of dimer geometries, contrary to the significant prevalence of the antiparallel arrangement suggested in previous works^30^,^31^.

Nevertheless, fixed ξ and θ alone do not unambiguously define individual dimer arrangements due to the asymmetric structure of AmB (Fig. 1) that allows different relative orientations of the mycosamine moieties. To fully characterize the geometry of AmB dimers, we computed the angle φ between the second principal axes of the macrolide ring (marked as AII in Fig. 1, bottom panel), thus distinguishing geometries in which the two mycosamine groups pointed in the same or opposite directions. We used an analogous procedure to calculate the free energy as a function of ξ and φ. In the profile in Fig. S1, it can be seen that dimers, in both parallel and antiparallel orientations, tend to assume a symmetric arrangement with the mycosamine moieties pointing in opposite directions, as φ is more often found close to 180 than to 0°. In particular, the highest-populated minimum II corresponds to a highly symmetric head-to-tail arrangement, where the mycosamine moiety acts as a lid over the other monomer’s tail group (see Fig. 2C).

### AmB monomers associate rapidly and easily flip between two equiprobable arrangements

The free energy profile in Fig. 2A indicates that AmB monomers aggregate in a barrierless, diffusion-controlled process, and are already attracted to each other at ξ = 20 Å, when they form a contact in an end-to-end orientation. From the profile, we estimated the rate constant for association, k_on_, to be 5·10^8 ^M^−1^·s^−1^ (for details see Supplementary Information), which is at least 2 orders of magnitude faster than membrane insertion^32^. This finding suggests that AmB dimers, and possibly higher oligomers as explained further in text, are fully equilibrated before entering the membrane.

Figure 2B reveals that for the intermediate separation distance, in the range of 10–14 Å, the free energy surface is slightly skewed and tends to decrease for higher values of θ, so that the free energy basin corresponding to the antiparallel dimer is noticeably broader in the ξ dimension. This suggests that aggregating AmB molecules may have a tendency to initially assume an antiparallel orientation. Indeed, all three additional unrestrained simulations starting from separated monomers (represented as white, red and black dots in Fig. 2B) followed a similar pathway and ended up forming antiparallel dimers. This additionally emphasizes the relevance of systematic sampling of the reaction coordinate space for the correct estimation of the free energy landscape and binding affinities.

Therefore, the formation of parallel dimers shall depend on the flipping rate, i.e., the rate of the antiparallel-parallel transition. From the free energy profile, we estimated the mean first passage time to be equal to 200 ns (corresponding to a rate constant of 5·10^6 ^s^−1^; see Supplementary Information for a detailed description of the model used). In fact, in three umbrella sampling windows such a transition occurred spontaneously, as could be expected from the flipping time scale derived from the free energy profile.

One might assume that parallel dimers will insert into the membrane more easily due to the polar heads (see Fig. 1A) being on the same end of the dimer, and thus the parallel dimer itself being more amphipathic than an antiparallel one. If we take into account that dimers are believed to differ from monomers in toxicity profile^22^,^23^, and that this difference probably stems from different affinity for the membrane, we can therefore speculate that AmB derivatives characterized by a more stable antiparallel dimers would exhibit different, possibly lower, toxicity than AmB alone.

### AmB dimer formation is dominated by hydrophobic forces

**Figure 3:**
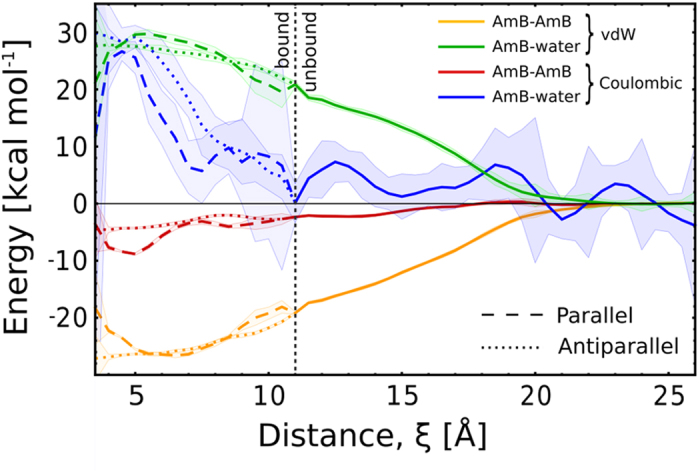
Individual contributions to the enthalpy of dimerization of AmB. Shaded areas illustrate the standard error for estimated energies. For the bound state (ξ < 11 Å), parallel and antiparallel dimers were considered separately.

To investigate the molecular determinants of the stability of AmB dimers and the equilibrium between the identified dimer geometries, we used the reweighted umbrella sampling data to calculate the individual contributions to the dimerization energy. As clearly visible from Fig. 3, van der Walls interactions between the AmB molecules stabilize the dimers by ca. 25 kcal/mol (orange lines). However, the overall stabilizing AmB-AmB interaction is noticeably weaker than the enthalpic cost of desolvation (green and blue lines). This clearly indicates a hydrophobic nature of the association, as the remaining stabilizing effect of 20–30 kcal/mol – the difference between the enthalpic terms and the dimerization free energy (Fig. 2A) – can only be attributed to the release of interfacial water to the bulk upon dimerization.

The hydrophobic origin of AmB dimerization finds further support in an analysis of hydrogen bonds formed by the two monomers. As shown in Fig. S2A, the loss of 1–3 water-AmB hydrogen bonds is not compensated for by a corresponding formation of new intermolecular AmB-AmB h-bonds (inset), but correlates with a smooth decrease of the solvent-accessible surface area (SASA) of AmB molecules (Fig. S2B), as could be expected for a hydrophobically driven association. It is worth noting that while the hydrophobic SASA obviously decreases during dimerization, a dimer has larger hydrophobic surface than a monomer (see Fig. S2B, inset), suggesting that – counterintuitively – the thermodynamic driving force for membrane insertion can be larger for a single dimer than for a single monomer. Provided that the thermodynamic preference of AmB monomers for ergosterol-rich membranes is relatively low, this marked change in affinity can contribute to the loss of specificity for fungal cells.

Interestingly, as seen from the decomposed dimerization energies (Fig. S3), the Coulombic interaction of the two large dipoles of the head groups approaching each other in the parallel geometry is stabilizing only at very close distances (<6 Å) that correspond to ca. 20% of dimers’ population, and destabilizing at intermediate separation, where the two carboxyl groups come close to each other. Moreover, even the enthalpic advantage is counterbalanced by the energetic cost of desolvation of ca. 20 kcal/mol, as seen in Fig. 3 (dashed red/blue lines). This further demonstrates that the electrostatic attraction of the polar groups is mostly irrelevant for the association of AmB monomers, even though it possibly modulates the parallel-antiparallel equilibrium.

Yet, while the association is dominated by hydrophobic attraction, the calculated change in SASA is quite similar, regardless of the monomer orientation (Fig. S2B). This suggests that the hydrophobic driving force should be similar for all orientations, and the relative stability of individual dimer arrangements should depend on direct intermolecular interaction between the monomers. The decomposed energy profiles in Fig. S3 allow to distinguish the dominant enthalpic factors that modulate the stability of individual dimer geometries. In particular, the parallel geometry marked as IV is stabilized by a favorable contact of the two polyol moieties, while in the one marked as III the polyol remains in favorable direct contact with both the polyene chain and the polar head. This clear distinction between dominant driving forces is lost in the antiparallel geometry, suggesting a smoother transition between geometries I and II.

In addition, our simulations indicate that the formation of the symmetric antiparallel dimer is often impaired by a steric clash between the methyl groups of the mycosamine head and hydrophilic tail. It is therefore plausible that modifications of the steric properties in these regions could stabilize the antiparallel geometry, yielding – as proposed above – dimers that do not easily enter the membrane and therefore can help avoid the toxic side-effects.

### Circular dichroism spectra reveal competing contributions of individual dimer geometries

**Figure 4:**
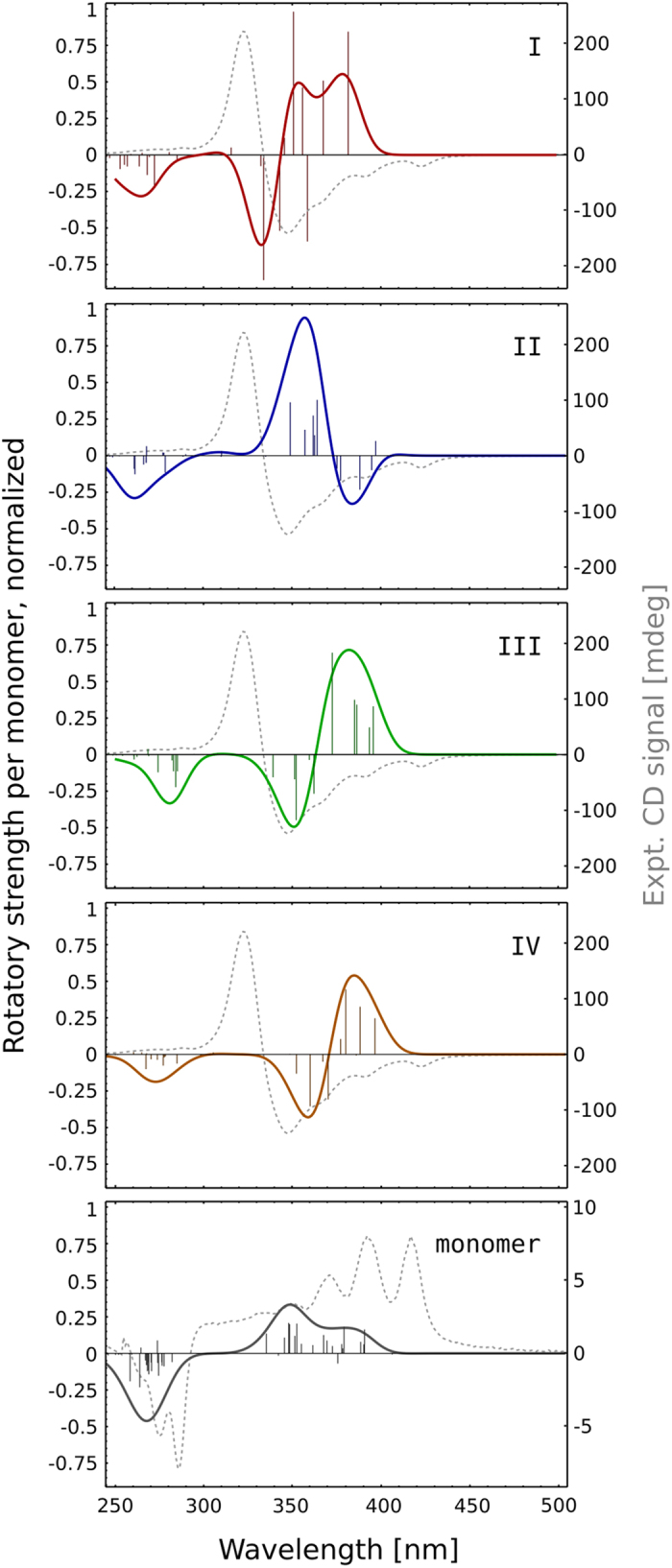
Circular dichroism (CD) spectra obtained by averaging over selected dimer (I-IV, according to Fig. 2B) and monomer geometries. The vertical lines correspond to individual excitations, which were convolved with a Gaussian function to simulate the continuous spectrum (solid line). Intensities are normalized to correspond to a single AmB molecule, and scaled to reflect the relative populations of dimer geometries. The gray dashed line corresponds to the experimental CD signal, taken from a recent study by Starzyk *et al.*^29^.

As researchers often relied on circular dichroism (CD) spectra in measuring the extent of AmB dimerization, we calculated the CD spectra of both AmB monomers and dimers (see Methods for a detailed description) to allow for a direct comparison with experimental results. In particular, this approach enabled us to assign specific CD signatures to individual dimer arrangements (I–IV), and thus to potentially relate the shape of the spectrum obtained experimentally to dimer geometries that prevail in AmB oligomers. The resulting spectra in Fig. 4 show a significant enhancement of the CD signal upon association, and the exciton doublet pattern expected for the dimerization of two optically active chromophores. Quite interestingly, out of the 4 considered arrangements (I-IV), only the antiparallel dimer labeled as II yielded spectra that – although shifted – are in qualitative agreement with the experimental data (here taken from a recent work by Starzyk *et al.*^29^), while both parallel geometries produced an inverted signal. Notably, the geometry II is also the most populated one (32%) in the dimeric state. This suggests that in higher-order oligomers, this particular arrangement of neighboring monomers can be prevailing due to a thermodynamic selection. However, the CD signal intensities of the dimers, albeit higher than in case of monomers, do not account for the enhancement of intensity observed in the spectroscopic studies, indicating that the structures observed experimentally could be higher oligomers or polymolecular aggregates of asymmetric structure, rather than sole dimers.

### Formation of higher oligomers inhibits monomer flipping and promotes further aggregation

To provide insight into the first steps of formation of higher AmB oligomers, we simulated the spontaneous association of pairs of AmB dimers, starting from all favorable dimer geometries identified in our dimerization simulations: parallel/parallel, parallel/antiparallel and antiparallel/antiparallel (see Methods for details). Tetramers were chosen as model intermediates of further oligomerization based on recent suggestions concerning their possible relevance for the channel-forming activity of AmB^29^,^33^. It should be noted here that the enormous configuration space available for tetramers cannot be sampled adequately with relatively short equilibrium simulations, which allows only for a qualitative discussion of the behavior of oligomers.

**Figure 5:**
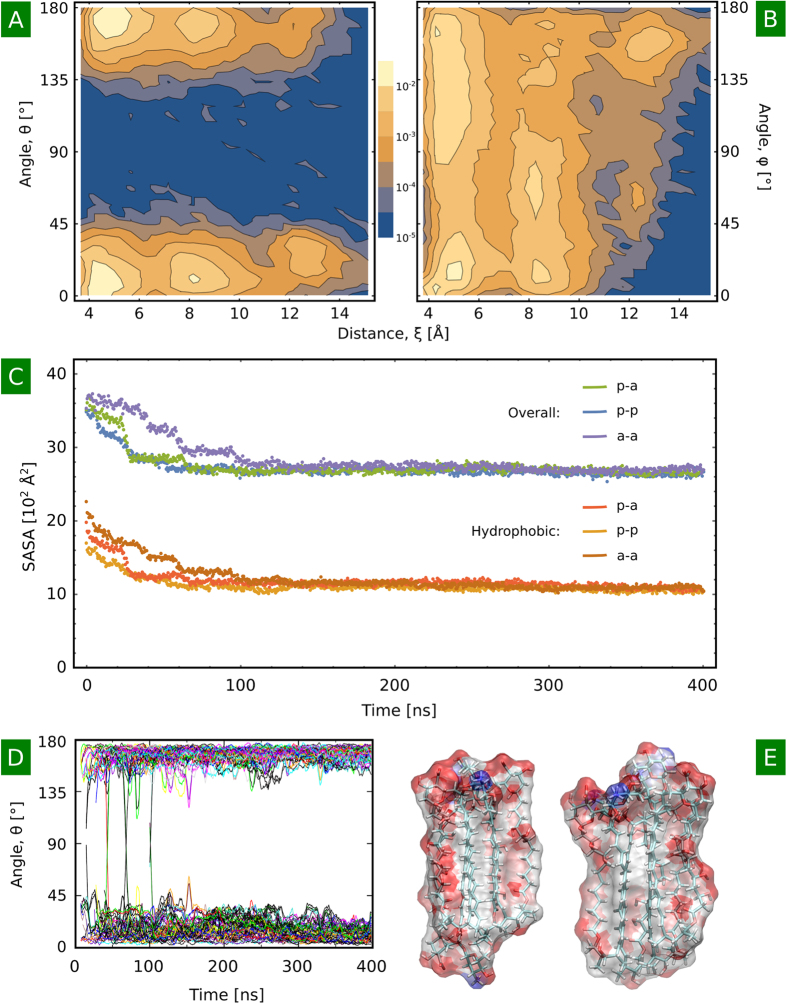
Spontaneous association of AmB dimers into tetramers. (**A,B**) Distribution of geometric parameters describing the relative orientation of AmB monomers in tetrameric structures, with coordinates ξ, θ and φ defined as previously. (**C**) Overall and hydrophobic solvent accessible surface area of AmB tetramers, in each case averaged over 6 trajectories of the parallel-antiparallel (p-a), parallel-parallel (p-p) and antiparallel-antiparallel (a-a) dimer pairs. (**D**) Time-evolution of intermonomer angle θ during the spontaneous aggregation of dimers. (**E**) Large hydrophobic patches on the surface of AmB tetramers indicate their high propensity to higher-order aggregation.

An analysis of the geometry of AmB tetramers formed in our simulations indicates that, although all monomers aligned along a common axis, assuming values of θ either close to 0° or to 180° (see Fig. 5D and Fig. S4), no clear propensity for a particular arrangement – either parallel or antiparallel – could be observed. Tetramers were characterized by an even larger structural heterogeneity than in case of dimers, as seen in the distribution of geometric coordinates ξ, θ and φ differing noticeably from that of dimers (see Fig. 5A,B). This indicates a lack of clear selection of the structures that yielded experimental-like CD signals in the dimeric form. Moreover, in the concentration of AmB used in our simulations (ca. 21 mM), the observed oligomerization occurred rapidly – on average taking 31 ns ± 24 ns – and all dimers formed tetramers within 100 ns (see Fig. 5C,D). Notably, while in 4 dimers the relative monomer orientation switched prior to association, no flipping was observed in the 18 tetramers in the remaining simulation time, until 400 ns. On the contrary, the time evolution of angles θ (see Fig. 5D) indicates that tetramers are becoming more stable during the course of a simulation, suggesting that higher oligomers trap the monomers in their initial orientation and inhibit flipping. Since we observed no preference for a particular arrangement, and given that parallel and antiparallel dimer geometries are roughly equiprobable, our results suggest that AmB molecules in higher oligomers, such as tetramers, align in parallel and antiparallel manner with roughly equal probability. Finally, it is worth noting that the solvent-exposed hydrophobic surface of dimers and tetramers is virtually the same (ca. 900 Å^2^) and larger than that of a monomer (ca. 650 Å^2^), indicating again that dimers and tetramers can have larger affinity for membrane insertion than monomers (see Fig. S2B and Fig. 5C,E).

## Conclusions

In the present work, we used molecular dynamics simulations to study the association of AmB molecules in an aqueous environment. Our free energy calculations indicate that dimers begin to form already in the therapeutic, sub-micromolar concentrations, and that their formation is a barrierless, diffusion-controlled process. The populations of parallel and antiparallel dimers are roughly equal (to within 6%), with a noticeable tendency to arrange in a symmetric manner. While AmB dimers turn out to be flexible and dynamic – with a sub-microsecond parallel-antiparallel flipping rate – the formation of tetramers significantly inhibited large structural rearrangements within the oligomer.

Moreover, the results of circular dichroism quantum chemical calculations reveal that individual stable dimer geometries yield quite different signature signals, but the resulting spectrum, when compared with that of a monomer, does not account for the spectral shift observed experimentally. This suggests that the shift, used to experimentally pinpoint the onset of oligomerization, results from the formation of higher oligomers, and not dimers alone.

As evidenced by previous studies, the selectivity of AmB depends on the oligomeric state of drug molecules: at low concentrations, when only AmB monomers exist in solution, the drug has a greater tendency to enter ergosterol-rich (fungal) membranes, while cholesterol-rich (mammalian) membranes remained impenetrable until higher drug concentrations. It is therefore plausible that the selectivity of the drug stems from the presence of monomers, and oligomerization abolishes the distinction between fungal and mammalian membranes and leads to the toxic side effects of AmB. Our results indicate that this dependence can partially stem from a large hydrophobic solvent-accessible surface area (SASA) of AmB oligomers: despite the laterally amphiphilic character of AmB (hydrophobic polyene and hydrophilic polyol chains), dimers and tetramers retained large hydrophobic SASA upon association. We also note that the quick interconversion between individual arrangements of dimers, with several roughly equiprobable stable geometries, can additionally facilitate the passage of dimers through a complex membrane-water interface.

## Methods

### Molecular dynamics simulations

We performed a series of molecular dynamics simulations of two amphotericin B molecules in an aqueous environment. All energy minimizations and molecular dynamics simulations of dimers were performed using NAMD^34^. Simulations of pairs of dimers were performed in Gromacs^35^, using the same set of force field and run parameters. Dimers were placed in a 6.25 × 6.25 × 6.25 nm cubic box containing 8153 TIP3P water molecules and 23 Na^+^ and 23 Cl^−^ ions, corresponding to physiological ion concentration of 0.15 mol/dm^3^. Pairs of dimers were placed in a cubic box of 6.8 × 6.8 × 6.8 nm, along with 9942 TIP3P water molecules and 29 Na^+^ and 29 Cl^−^ ions, corresponding to the same ion concentration. The pressure was kept constant at 1 bar using the Langevin piston method^36^. The temperature was maintained at 300 K using the Langevin thermostat. Particle-mesh Ewald summation^37^ was used to calculate long-range electrostatic interactions, and the corresponding short-range part was computed with a direct-space cutoff of 1.0 nm. The Lennard-Jones potential and forces were smoothly switched off between 1.0 and 1.2 nm. The SHAKE algorithm^38^ was used to constrain the length of covalent bonds involving hydrogen atoms, except for water, for which the SETTLE algorithm^39^ was applied. This allowed for a time step of ∆t = 2 *fs* to be used to integrate the equations of motion with the velocity Verlet algorithm.

For the antibiotic molecule, the same parameter set was used as in the number of previous studies^17^,^40^,^41^. AmB molecules were in the zwitterionic form, i.e., with deprotonated carboxyl and protonated amino groups. The free energy profiles for the dimerization of AmB monomers in the aqueous environment were obtained using the umbrella sampling (US) method^42^ and the Weighted Histogram Analysis Method (WHAM)^43^. In our simulations, we divided the interval 4.0 < *ξ <* 25.0 Å into 17 umbrella sampling windows. For each window and two arrangements of the dimer (parallel and antiparallel), 200 ns of MD trajectory was generated, yielding a total simulation time of 6.8 μs. The initial structures of stable AmB dimers were taken from a recent study^29^. The starting geometries for each US window were obtained from two steered MD simulations, in which the AmB molecules forming an antiparallel or parallel dimer were pulled away from each other up to the distance 25 Å. Pairs of dimers were placed in an orthogonal configuration in such a way that the PBC images in the XY plane were equally spaced. For each possible pair of dimers (parallel-parallel, parallel-antiparallel, antiparallel-antiparallel), six 400-ns equilibrium trajectories were generated, starting from different initial configurations, yielding a total simulation time of 7.2 μs.

### Calculation of circular dichroism spectra

For the circular dichroism (CD) calculations, representative structures were extracted from the umbrella sampling trajectories. 20 structures of the monomer were obtained from the last umbrella sampling window (*ξ*_*eq*_ *=* 25.0 Å), where the AmB molecules were separated. From each minimum labeled as I-IV in Fig. 2B, 5 structures of dimers were extracted, also yielding a total of 20 dimeric AmB structures. Electronic circular dichroism spectra were calculated for each structure using the TD-DFT method as implemented in the Gaussian software^44^. The ωB97X exchange-correlation functional with empirical dispersion scheme (ωB97XD) was employed^45^, along with the correlation-consistent pVDZ Dunning basis set^46^. The polarizable continuum model (PCM) was used to account for the aqueous environment. The obtained spectra were Gaussian smoothed by convolving them with a Gaussian function and scaled so that the intensities (1) correspond to a single AmB molecule and (2) represent the relative populations of the dimers’ geometries (I–IV).

### Calculation of thermodynamic and kinetic parameters

The standard free energy of AmB dimerization was calculated according to the following formula:


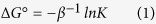


where *β* is the inverse temperature *(k*_*B*_*T)*^*−1*^ and the unitless dimerization constant *K* is obtained by integrating the free energy profile over the dimeric state^47^:


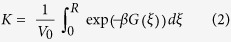


where *V*_*0*_ is the standard volume (1661 Å^3^) corresponding to the standard concentration of 1 M and R is the upper distance boundary defining the dimeric state (here 11 Å). Note that because the free energy profile *G(ξ)* is taken directly from the WHAM procedure, the probability density *ρ(ξ)* ~ *exp(−βG(ξ))* is already integrated over the angular coordinates.

The on-rate was obtained as a solution to the steady-state Smoluchowski equation from the following formula^48^:


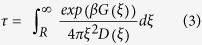



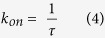


where *G(ξ)* is the free energy profile and *D(ξ)* is the diffusion coefficient, calculated at mean values of intermolecular distance *ξ* in each umbrella sampling window as proposed by Hummer^49^:


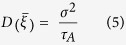


with *σ^2^* being the variance of the distribution and *τ*_*A*_ the autocorrelation time. The values of the coefficient were interpolated at intermediate distances, and extrapolated beyond the sampled interval as a constant.

The mean first-passage time (MFPT) from a parallel configuration at angle *θ*_*0*_ to an anti-parallel configuration at angle *θ*_*1*_ was calculated as proposed by Zwanzig by using the formula^50^:


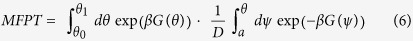


with *a* being the position of the reflecting barrier placed at *θ* = 0° or *θ* = 180° and *D* being the rotational diffusion coefficient, computed according to the method by Kinosita and Ikegami for a wobbling-in-a-cone model^51^. In the model, the apparent relaxation time was calculated as the autocorrelation time for the second Legendre polynomial of the cosine of the angle formed by two monomers, and the sigma parameter was obtained by fitting a gaussian function to the entropy-corrected angular distribution. The value of D was estimated as equal to 3.8 · 10^12 ^deg^2^ s^−1^.

## Additional Information

**How to cite this article**: Zielińska, J. *et al.* Thermodynamics and kinetics of amphotericin B self-association in aqueous solution characterized in molecular detail. *Sci. Rep.* **6**, 19109; doi: 10.1038/srep19109 (2016).

## Supplementary Material

Supplementary Information
