# Supplementary material for: Thermodynamics and kinetics of amphotericin B self-association in aqueous solution characterized in molecular detail
Source: Sci Rep. 2016 Jan 8;6:19109. doi: 10.1038/srep19109 (PMC4705489; doi:10.1038/srep19109)
Supplement: Supplementary Information [file srep19109-s1.pdf]

Thermodynamics and kinetics of amphotericin B self-association in aqueous solution characterized in molecular detail

Joanna Zielińska[1,*] , Miłosz Wieczór[2,*], Tomasz Bączek[1], Marcin Gruszecki[3,‡], Jacek Czub[2, †]

[1]Department of Pharmaceutical Chemistry, Medical University of Gdańsk, Gdańsk, Poland

[2]Department of Physical Chemistry, Gdańsk University of Technology, Gdańsk, Poland

[3]Department of Radiological Informatics and Statistics, Medical University of Gdańsk, Gdańsk, Poland

‡mgruszecki@gumed.edu.pl

†jacczub@pg.gda.pl

*these authors contributed equally to this work

Supplementary Information

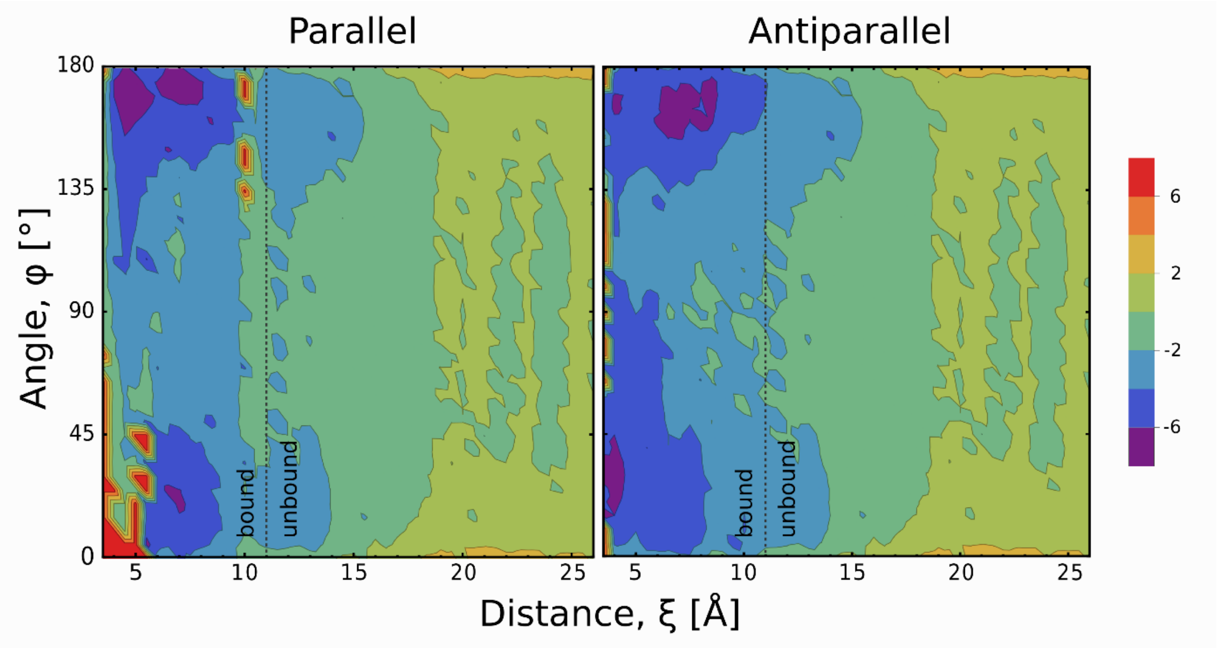

Supplementary Figure S1. 2D free energy profiles of two AmB molecules in a parallel and antilparallel geometry, as a function of the interchromophore distance, ξ, and the angle between the 2nd main axes of both monomers, φ (see Fig. 1 for a depiction of the coordinates). The minima are marked as I-IV according to Fig. 2B.

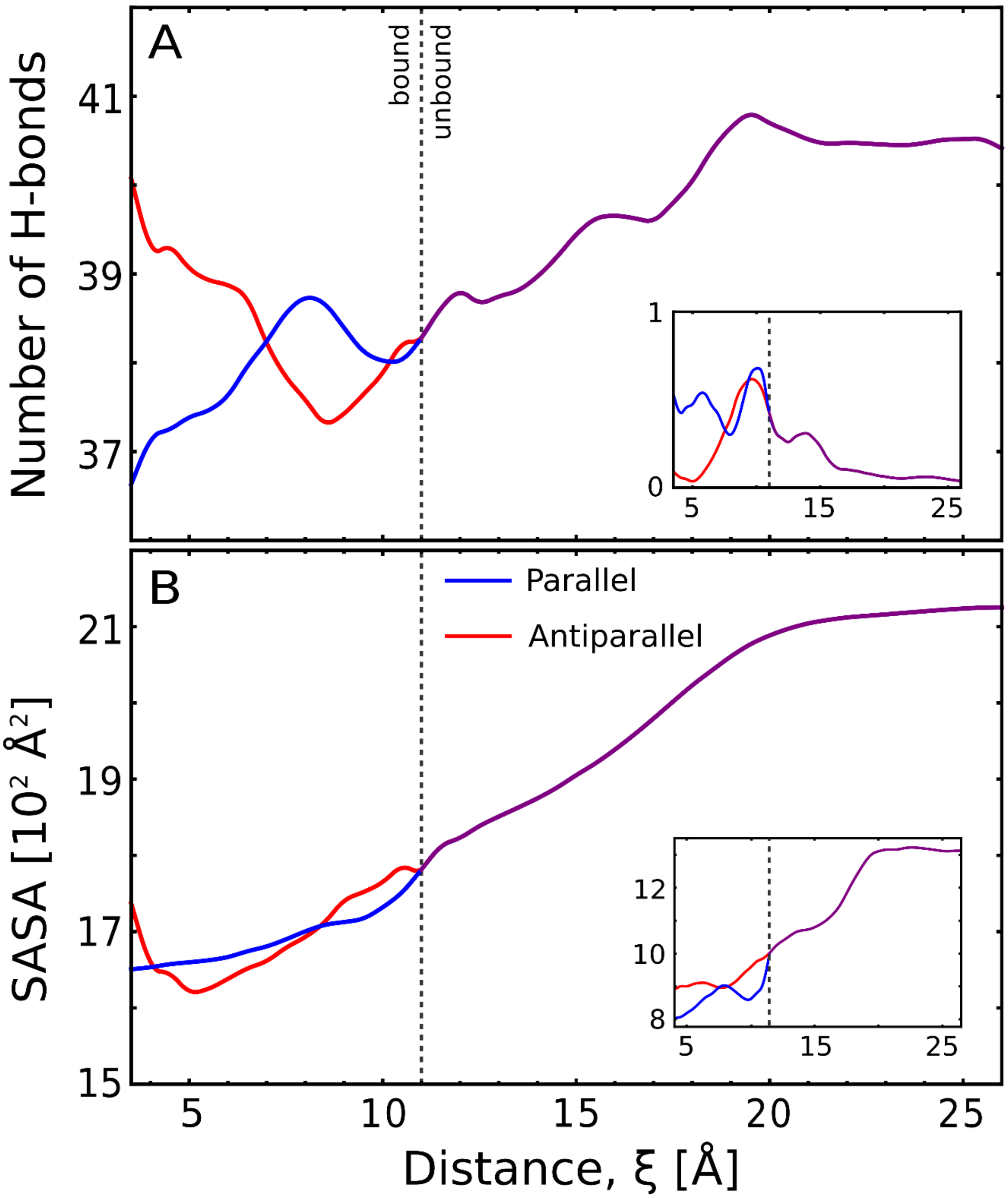

Supplementary Figure S2. A: Number of hydrogen bonds formed between AmB and water molecules (main panel) and two AmB monomers (inset) as a function of the intermonomer distance ξ. A hydrogen bond was defined by a D-A distance threshold of 3.5 Å and a D-H-A angle threshold of 40°, where D, A and H are the donor, acceptor and hydrogen atoms, respectively. **B:** Overall and hydrophobic (inset) solvent accessible surface area as a function of ξ. For the bound state (ξ < 11 Å), parallel and antiparallel dimers were considered separately.

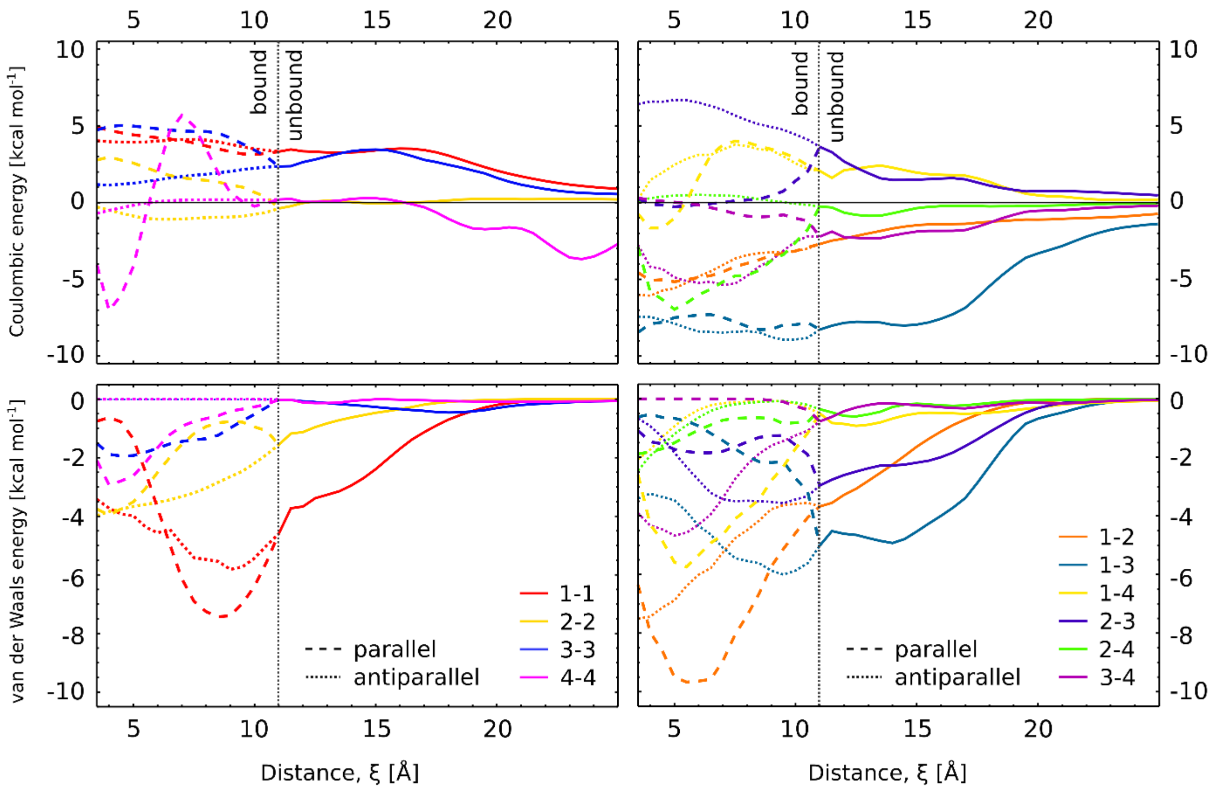

Supplementary Figure S3. Decomposed intermolecular interaction energies (Coulombic and van der Waals) between the respective parts of AmB monomers. Contributions are labeled according to the numbering scheme in Fig. 1. (polyol chain, 1; polyene chain, 2; polar tail, 3; mycosamine head, 4).

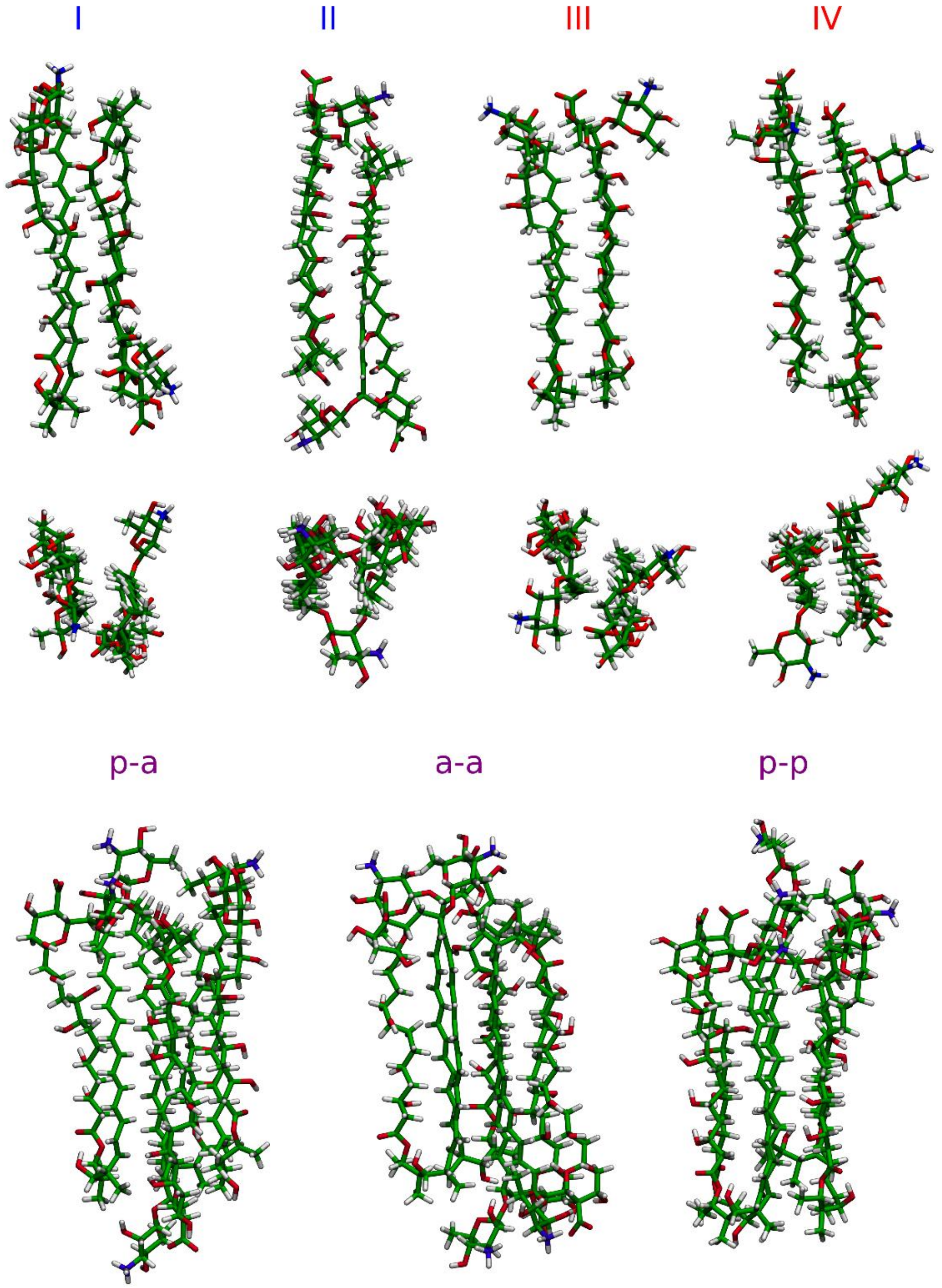

Supplementary Figure S4. High-resolution visualisation of identified stable dimer structures (I-IV) and representative tetramer structures (p-a: parallel-antiparallel; a-a: antiparallel-antiparallel; p-p: parallel-parallel).
